# Effect of RAS inhibitors on retinal microvasculature in DKD using AI-based ultra-wide-field images

**DOI:** 10.3389/fendo.2026.1840180

**Published:** 2026-06-26

**Authors:** Yan Liu, Xinyu Zhao, Haiyun Wang, Jie Ma, Peng Xia, Xingwang Gu, Youxin Chen, Limeng Chen

**Affiliations:** 1 Department of Nephrology, Peking Union Medical College Hospital, Chinese Academy of Medical Science & Peking Union Medical College, Beijing, China; 2 Beijing Key Laboratory of Molecular Pathology Diagnosis and Translational Research on Rare and Complex Urinary System Diseases, Beijing, China; 3 Department of Ophthalmology, Peking Union Medical College Hospital, Chinese Academy of Medical Science & Peking Union Medical College, Beijing, China

**Keywords:** artificial intelligence model, diabetic kidney disease, diabetic retinopathy, retinal microvascular parameters, ultra-wide-field fundus images

## Abstract

**Background:**

Diabetic kidney disease (DKD) and diabetic retinopathy (DR) represent two major microvascular complications of diabetes mellitus (DM). Previous studies have suggested that renin-angiotensin system inhibitors (RASi) exert protective effects on both DKD and DR. However, their specific impact on retinal microvascular parameters (RMPs), as well as the association between changes in fundus microvasculature and alterations in renal clinical parameters, remains unclear. This pilot study aimed to quantitatively assess the short-term effects of RASi on retinal microvasculature in patients with DKD using an artificial intelligence (AI)-based analysis of ultra-wide-field (UWF) fundus images.

**Methods:**

In this prospective cohort study, 27 patients with DKD were enrolled between July 2023 and September 2024. UWF fundus images were acquired at baseline and 12 weeks after initiation of RASi therapy. A validated deep learning AI model was employed to segment retinal vessels and quantify RMPs, including fractal dimension (D*f*) and tortuosity (TORT), in both the central and peripheral retinal regions. Statistical analyses for pre- and post-treatment comparisons were performed using a linear mixed-effects model with patient ID as a random intercept. or a Wilcoxon signed-rank test, as appropriate. Changes in these parameters post-treatment were analyzed and correlated with alterations in clinical renal indicators.

**Results:**

Among the enrolled patients, 21 patients (77.8%) were male, with a mean age of 55.7 ± 14.2 years, a mean diabetes duration of 9.9 ± 6.6 years, a baseline estimated glomerular filtration rate (eGFR) of 73.7 ± 18.5 mL/min/1.73 m², and a median proteinuria of 0.57 (0.25, 0.97) g/24h. Fifteen patients (55.6%) had diabetic retinopathy (DR). After 12 weeks of RASi treatment, significant decreases were observed within the UWF images in venous D*f* (adjusted *p* = 0.0389) for the overall cohort, and venous TORT (adjusted *p* = 0.0496) for No-DR and NPDR groups. These significant changes were not observed in parameters derived from the central retinal region.

**Conclusion:**

RASi therapy might be associated with retinal peripheral venous alterations, providing clues to a vascular- and topography-specific therapeutic response and shedding light on a potential imaging biomarker for diabetes management.

## Introduction

Diabetes mellitus (DM) is a prevalent global chronic metabolic disorder with a steadily rising incidence. Its associated microvascular complications, including diabetic kidney disease (DKD) and diabetic retinopathy (DR), pose significant threats to human health and quality of life (1, 2). Clinically, DKD and DR often occur synchronously (3), which is closely related to their shared pathological basis (4). Retinal blood vessels can serve as an ideal window for observing changes in systemic microvasculature, including renal blood vessels (5).

The landmark clinical trial that first demonstrated the renoprotective effects of RASi independent of blood pressure reduction in DKD was published in the *New England Journal of Medicine* (NEJM) in 1993, with captopril (an angiotensin-converting enzyme inhibitor, ACEI) adopted in the trial (6). The subsequent RENAAL and IDNT trials in 2001 further confirmed the renoprotective benefits of RASi with angiotensin receptor blockers (ARBs) (7, 8). In the clinical management of DKD, RASi, including ACEIs, ARBs, and renin inhibitors, have been confirmed by numerous clinical studies to play a definite role in reducing urinary protein excretion and improving renal prognosis (9). In addition, the UKPDS, published in 1998, established intensive glycemic control as a cornerstone for preventing microvascular complications (10); and the EUCLID study, also in 1998, was the first large-scale randomized controlled trial (RCT) to confirm the protective effect of RASi against DR (11). Subsequently, accumulating evidence indicates that RASi also exerts a protective effect on DR by inhibiting retinal vascular permeability, oxidative stress, and abnormal neovascularization (12), which is closely related to the crucial role of RAS overactivation in the occurrence and development of DR.

However, despite the well-documented protective effects of RASi on both DKD and DR, the dynamic process of fundus vascular morphological changes following RASi administration remains unclear. In particular, the correlation between these vascular changes and alterations in clinical indicators has yet to be elucidated. UWF fundus imaging expands the scope of fundus observation and enhances the acquisition of peripheral retinal information, while artificial intelligence (AI)-based analysis deepens the depth of fundus evaluation by enabling accurate quantitative analysis and providing precise and reproducible biomarkers. Therefore, this exploratory study aims to quantitatively assess the retinal microvascular parameters (RMPs) in a cohort of DKD patients with a consistent duration of RASi intervention, using UWF fundus images and AI analysis. Furthermore, we intend to explore the correlation between these RMPs and clinical indicators, thereby providing new theoretical foundations and clinical evidence for the individualized treatment of patients with DKD and DR.

## Methods

### Study design and participants

This prospective, single-center study was approved by the institutional ethical review board of Peking Union Medical College Hospital (Ethical No: KS2022438). Between July 2023 and September 2024, adults aged 18–75 years with a clinical diagnosis of diabetic kidney disease (DKD) were consecutively enrolled in our study, and written informed consent was obtained from all patients. The inclusion criteria further included estimated glomerular filtration rate (eGFR) ≥45mL/min/1.73m², urinary albumin-to-creatinine ratio (ACR) ranging from 30 mg/gCr to 3000 mg/gCr (inclusive of the lower limit), and no history of hyperkalemia. Patients with concurrent other primary or secondary kidney diseases, acute renal insufficiency, or nephrotic syndrome were excluded. Additionally, those with a recent history of severe cardio-cerebrovascular events, active infections, surgery, tumors, or other relevant comorbidities were also excluded from the study cohort. None of the patients had received RASi for at least 4 weeks prior to enrollment, including ARBs, ACEIs, renin inhibitors, and others; all met the study’s inclusion and exclusion criteria. Since this was an exploratory pilot study, no formal sample size calculation based on power analysis was performed. The sample size was determined by practical considerations of recruitment feasibility over the study period. Finally, 27 patients were enrolled. This sample size fell within the typical range (12–35 participants) recommended for exploratory studies (13, 14) and was also consistent with previous similar exploratory investigations (15, 16). Clinical baseline data and fundus images of the patients were collected at enrollment, after which RASi treatment was initiated. The medications included ARBs (eight patients with Valsartan 80 mg once daily) and renin inhibitors (nineteen patients with Sitokiren 50mg-200mg once daily). Clinical data and fundus photography images were re-examined 12 weeks later, and fundus images obtained before and after medication were analyzed using a validated AI model (17). No control group was included in this study.

### Clinical parameters

When the patients were enrolled in our study, case notes and laboratory records were collected from the Hospital Information System. The glomerular filtration rate (GFR) was estimated using the Creatinine- and Cystatin C-based Chronic Kidney Disease Epidemiology Collaboration (CKD-EPI) equation (2021) (18).

Male:κ=0.9, α=-0.207, eGFR=135×min(Scr/κ, 1)^α^×max(Scr/κ, 1)^-0.601^×min(CysC/0.8, 1)^-0.375^×max(CysC/0.8, 1)^-0.711^×0.995^age^

Female:κ=0.7, α=-0.248, eGFR=135×min(Scr/κ, 1)^α^×max(Scr/κ, 1)^-0.601^×min(CysC/0.8, 1)^-0.375^×max(CysC/0.8, 1)^-0.711^×0.995^age^×0.969

Where min indicates the minimum of Scr/κ or 1, and max indicates the maximum of Scr/κ or 1. Age in years, weight in kg; Scr, serum creatinine, in mg/dL, 1mg/dL=88.4µmol/L; CysC, serum cystatin C, in mg/L.

### Fundus examination protocol

Bilateral Optos UWF fundus images (Daytona, Optos PLC, Dunfermline, UK) were evaluated independently by two retinal specialists (XYZ and XWG) and categorized as either acceptable or unacceptable based on the exclusion criteria. Inter-observer discrepancies were quantified using kappa statistics, yielding a consistency rate of 95.4% (95% CI: 92.8-99.8%). If there was controversy between two retinal specialists, a final consensus was achieved through consultation with a senior retinal expert (YXC). The inclusion criteria were consistent with those in the clinical section, and the ophthalmic exclusion criteria included: 1) images with significant artifacts; 2) blurring due to vitreous hemorrhage, astrocytosis, or severe cataracts; 3) inadequate visualization of retinal vessels, optic disc, or other fundus structures; 4) history of vitreoretinal surgery; and 5) over- or underexposed images. The missing data were mainly due to poor image quality, and no further analysis was conducted on this part of the data.

### Segmentation and measurement of retinal vascular metrics in UWF images

**Figure 1:**
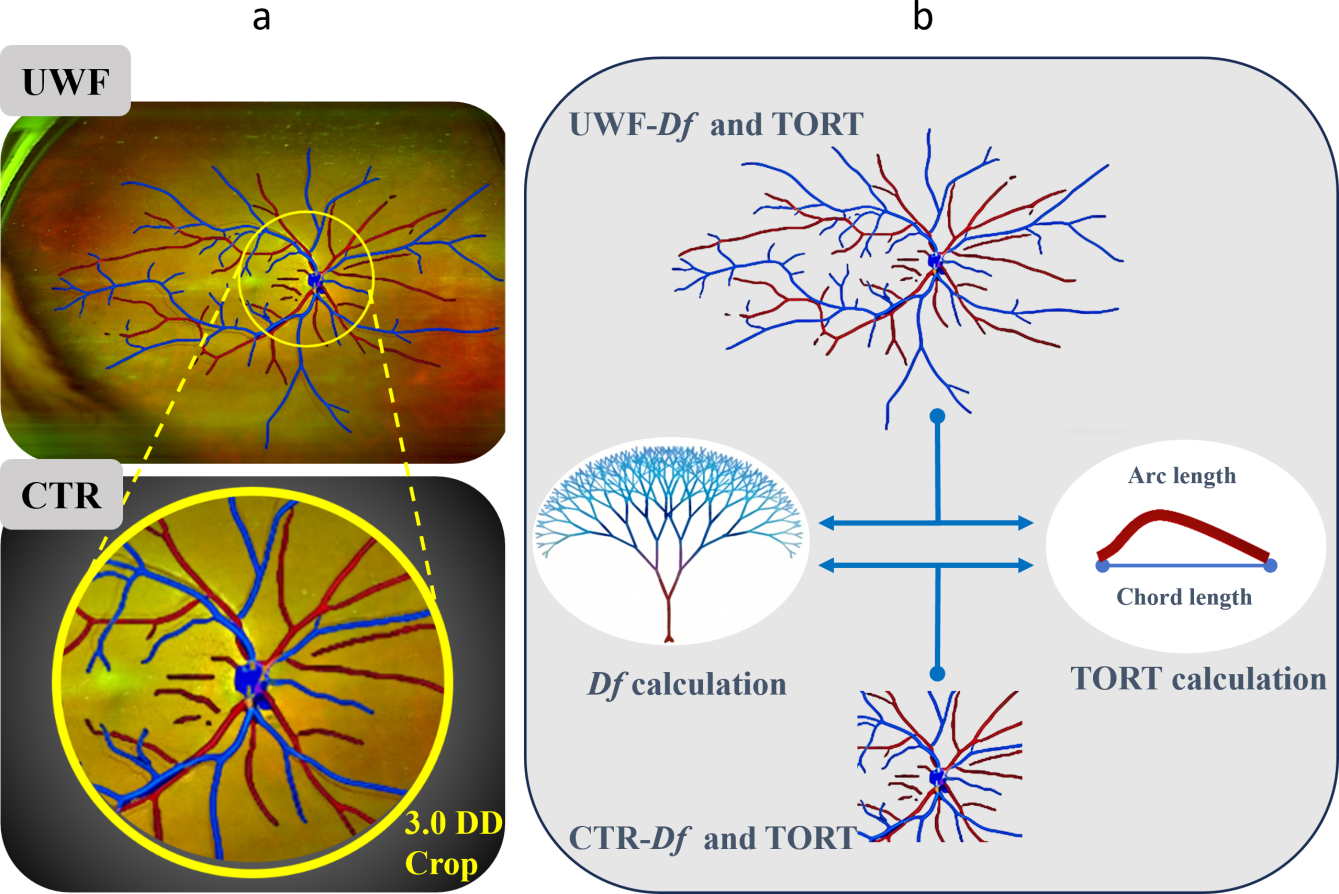
Schematic diagrams of ultra-wide-field (UWF) and optic disc central region (CTR) images, as well as the significance of fractal dimension (D*f*) and tortuosity (TORT). **(a)** An optic disc-centered circular region with a radius of 3 DD away from the optic disc was cropped from the UWF image to represent the central 50°fundus region (denoted as CTR). **(b)** The D*f* and TORT were calculated with an AI model in both UWF and CTR images.

Vessel segmentation was performed using a UNet++ architecture, as detailed in our prior publication (17), which employed an EfficientNet B5 encoder. The development and validation of this AI model were detailed in a previous article (19). The model produced three separate channels corresponding to arteries, veins, and optic discs, enabling fully automated measurement of retinal vascular parameters. Detailed information on the AI model from our previous study was as follows (17): Dataset Characteristics: Training set: 7,781 patients with 23,313 UWF images; External validation set: 1,352 patients with 3,226 UWF images, prospectively collected from 23 tertiary hospitals nationwide. The model had DICE scores of 0.54, 0.61, and 0.88 for arteries, veins, and optic discs, respectively. The AUC scores were 0.74 (95% CI: 0.70-0.79), 0.80 (95% CI: 0.76-0.83), and 0.94 (95% CI: 0.91-0.98), respectively. When the segmentation was limited in the posterior fundus region, the DICE scores were 0.64, 0.70, and 0.88, respectively, and the AUC scores were 0.83 (95% CI: 0.81-0.85), 0.86 (95% CI: 0.84-0.88), and 0.94 (95% CI: 0.91-0.98), respectively. The fractal dimension (D*f*) was derived via the box-counting method. Briefly, the vessel mask was partitioned into grids of side length r, and the count of grids covering vascular structures, denoted as N(r), was recorded. A linear regression of N(r) against r was fitted, with the slope representing the D*f*. Vessel curvature was quantified as simple tortuosity (TORT): each vessel segment was isolated from branching points, and the ratio of its actual length to the Euclidean distance between endpoints was computed. The mean ratio across all segments defined the TORT for the image. To examine associations between peripheral retinal regions and renal function, a central region (labeled as CTR) was extracted from each UWF image, approximating the 50°field of view of conventional fundus cameras (**Figure 1**). D*f* and tortuosity (TORT) were calculated for both the full UWF and CTR (denoted UWF/CTR-D*f* and UWF/CTR-TORT). Subgroup analyses were further conducted for arterial and venous metrics.

### Statistical analysis

SPSS 27.0 for Windows (SPSS Inc., Chicago, IL, USA) was used for the statistical data analysis. The Kolmogorov-Smirnov test was used to test the normality of the data. Normally distributed variables were expressed as mean **±** standard deviations. Non-normally distributed variables were reported as median values with quartiles. Categorical variables were described as percentages. A one-way analysis of variance (ANOVA) was conducted to compare the clinical and laboratory data among the three independent groups with different grades of DR. For ANOVA results demonstrating a statistically significant main effect (p<0.05), Tukey’s Honest Significant Difference (HSD) *post hoc* test was performed to identify specific pairwise group differences. For each RMP, homogeneity of variance was first assessed by Levene’s test. As the equal-variance assumption was satisfied for all 12 parameters, group differences were evaluated by one-way ANOVA; the pre-specified Welch’s ANOVA with Tamhane’s T2 procedure for unequal variances was therefore not required. The *p*-values were then adjusted using the Benjamini–Hochberg method to control the False Discovery Rate (FDR) at 0.05, and Tukey’s HSD *post hoc* tests were performed only for parameters that remained significant after FDR adjustment. Based on the results of normality testing, associations between clinical data and RMPs at baseline were quantified using Pearson’s (if normally distributed) or Spearman’s (if non-normally distributed) correlation coefficients, as appropriate. Paired *t*-test (if normally distributed) or Wilcoxon matched-pairs signed-rank test (if non-normally distributed) were used to compare the changes in clinical data of the same patient after RASi treatment. A linear mixed-effects model with patient ID as a random intercept was used to compare the changes in RMPs of the same patient after RASi treatment. The *p*-values were also adjusted using the Benjamini–Hochberg method to control the False Discovery Rate (FDR). For linear mixed-effects model, Cohen’s d, mean differences, and 95% CIs were reported. For Wilcoxon signed-rank tests, r, median differences, and 95% CIs were presented. The correlations between changes (Δ) in clinical data (ΔKidney) and changes in RMPs (ΔRMPs) were analyzed using Spearman’s rank correlation. Multivariable linear regression analysis was performed to assess the independent association between ΔRMPs and ΔKidney. A two-tailed *p* value less than 0.05 was considered statistically significant.

## Results

### Baseline clinical, laboratory, and ocular data

**Figure 2:**
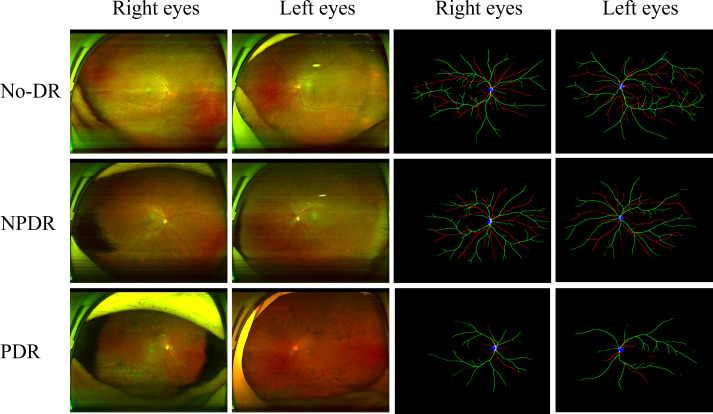
AI-based Segmentation and Quantitative Evaluation of Schematic Diagrams for Diabetic Retinopathy at Different Stages.

Twenty-seven patients with DKD were enrolled in our study, and the baseline data were presented in **Table 1**. There were 21 (77.8%) males, with a mean age of 55.7 ± 14.2 years at recruitment. The mean duration of diabetes mellitus was 9.9 ± 6.6 years, and 15 patients (55.6%) were complicated with diabetic retinopathy. The comorbidities included hypertension (77.8%), hyperlipidemia (77.8%), hyperuricemia (33.3%), and cerebral infarction (22.2%). At baseline, the average eGFR was 73.7 ± 18.5 mL/min/1.73m^2^ (range 45.5-115.8 mL/min/1.73m^2^), and the proteinuria was 0.57 (0.25, 0.97) g/24h (range 0.16-4.39 g/24h). The patients were divided into three groups: the non-diabetic retinopathy group (No-DR, n=12), the non-proliferative diabetic retinopathy group (NPDR, n=9), and the proliferative diabetic retinopathy group (PDR, n=6). Two patients from the No-DR and NPDR groups had data available for only one eye, due to poor image quality in the contralateral eye. The characteristic fundus images and AI segmentation maps of each group are shown in **Figure 2**. There were no significant differences in age or gender among the three groups. Similarly, no significant differences were observed in blood pressure, HbA1c, or serum lipid profiles at baseline. In addition, no significant differences were found in serum creatinine (Cr), serum cystatin C (CysC), or eGFR. The proteinuria in PDR group was significantly higher than that in the other two groups, as assessed by 24-hour urinary protein excretion (overall *p* = 0.035), urinary albumin-to-creatinine ratio (ACR) (overall *p* = 0.027), and urinary protein-to-creatinine ratio (PCR) (overall *p* = 0.020). Analyzed by the AI model, twelve RMPs were obtained (**Table 2**). Intergroup comparisons among the three groups revealed significant differences across 7 indicators: CTR-Artery-D*f*, UWF-Artery-D*f*, UWF-Artery-TORT, UWF-Vein-D*f*, CTR-D*f*, UWF-D*f*, and UWF-TORT. After BH-FDR correction, only four parameters remained statistically significant (CTR-Artery-D*f*, UWF-Artery-D*f*, CTR-D*f*, and UWF-D*f*; with adjusted *p* < 0.001, 0.005, and 0.006, respectively). Notably, all four RMPs were fractal-dimension metrics. The remaining three indicators that were nominally significant in the unadjusted analysis (UWF-Artery-TORT, UWF-Vein-D*f*, and UWF-TORT; with raw p=0.024, 0.034, and 0.026, respectively) no longer reached significance after correction (adjusted *p* = 0.053, 0.059, and 0.053, respectively). For all four robustly significant parameters, *post hoc* comparisons showed a consistent pattern: these RMPs were significantly lower in the PDR group than in both the No-DR and NPDR groups (all adjusted p<0.01; **Supplementary Table 1**), with no significant difference between No-DR and NPDR groups.

**Table 1:** Baseline demographic and clinical data in different grades of diabetic retinopathy (DR).

Characteristics	Total	No DR	NPDR	PDR	*P*-value
Demographic and clinical data					
No. of patients, %	27	12 (44.4%)	9 (33.3%)	6 (22.2%)	
No. of eyes, %	52	23 (44.2%)	17 (32.7%)	12 (23.1%)	
Males/females	21/6	9/3	7/2	5/1	0.931
Age, year	55.7 ± 14.2	54.3 ± 17.7	55.8 ± 13.5	58.3 ± 7.2	0.858
Systolic Blood Pressure, mmHg	130 ± 7	131 ± 5	130 ± 3	127 ± 8	0.529
Diastolic Blood Pressure, mmHg	81 ± 7	79 ± 9	83 ± 5	83 ± 7	0.520
Laboratory data					
SCr, μmol/L	78.1 ± 17.7	78.4 ± 19.0	81.3 ± 20.5	72.5 ± 10.6	0.654
CysC, mg/L	1.28 ± 0.33	1.21 ± 0.32	1.32 ± 0.32	1.37 ± 0.42	0.620
eGFR^a^, mL/min/1.73m^2^	73.7 ± 18.5	76.4 ± 19.0	71.2 ± 21.7	71.9 ± 13.9	0.802
HbA1c, %	7.38 ± 0.74	7.28 ± 0.87	7.56 ± 0.66	7.33 ± 0.62	0.695
Total Cholesterol, mmol/L	4.72 ± 1.28	4.85 ± 1.28	4.61 ± 1.46	4.62 ± 1.22	0.898
Triglyceride, mmol/L	2.80 ± 2.74	3.31 ± 3.02	2.78 ± 3.13	1.84 ± 1.24	0.584
Proteinuria, g/24h	0.57 (0.25, 0.97)	0.64 (0.32, 0.91)	0.38 (0.24, 0.49)	1.54^b^ (0.62, 2.49)	0.035^*^
PCR, mg/gCr	316 (153, 1520)	312.50 (114.50, 465.75)	286 (204, 308)	1634.5^c^ (828.50, 2033.25)	0.020^*^
ACR, mg/gCr	235 (108, 610)	191.5 (67.25, 358)	158 (128, 235)	962^b^ (406.75, 1852.50)	0.027^*^

No-DR, no diabetic retinopathy; NPDR, nonproliferative diabetic retinopathy; PDR, proliferative diabetic retinopathy; SCr, serum creatinine; CysC, Cystatin C; eGFR, estimated glomerular filtration rate; PCR, protein-to-creatinine ratio; ACR, albumin-to-creatinine ratio.

^a^ the eGFR was calculated according to the Creatinine- and Cystatin C-based EPI-CKD equation (2021) (18).

*p<0.05 one-way ANOVA.

^b^ Adjusted *p* < 0.05 vs. NPDR group, pairwise comparisons with Tukey’s HSD *post hoc* tests for multiple testing.

^c^ Adjusted *p* < 0.05 vs. No DR group, pairwise comparisons with Tukey’s HSD *post hoc* tests for multiple testing.

**Table 2:** Baseline retinal microvascular parameters (RMPs) in different grades of DR.

Retinal vascular metrics	Total (N = 27)	No DR (N = 12)	NPDR (N = 9)	PDR (N = 6)	RAW p-value	BH-FDR adjusted p-value
CTR-Artery-Df	1.328 ± 0.080	1.356 ± 0.063	1.350 ± 0.054	1.240 ± 0.081	<0.001**	<0.001^aa^
CTR-Artery-TORT	1.335 ± 0.080	1.333 ± 0.062	1.338 ± 0.095	1.333 ± 0.094	0.985	0.985
CTR-Vein-Df	1.322 ± 0.050	1.325 ± 0.047	1.328 ± 0.052	1.304 ± 0.054	0.416	0.503
CTR-Vein-TORT	1.399 ± 0.122	1.384 ± 0.107	1.389 ± 0.123	1.440 ± 0.145	0.419	0.503
UWF-Artery-Df	1.223 ± 0.136	1.258 ± 0.124	1.280 ± 0.084	1.079 ± 0.121	<0.001**	<0.001^aa^
UWF-Artery-TORT	1.354 ± 0.086	1.362 ± 0.068	1.384 ± 0.093	1.298 ± 0.089	0.024*	0.053
UWF-Vein-Df	1.304 ± 0.071	1.314 ± 0.074	1.322 ± 0.057	1.259 ± 0.065	0.034*	0.059
UWF-Vein-TORT	1.447 ± 0.098	1.440 ± 0.085	1.476 ± 0.098	1.421 ± 0.118	0.309	0.463
CTR-Df	1.472 ± 0.066	1.493 ± 0.059	1.486 ± 0.056	1.414 ± 0.061	0.001**	0.005^aa^
CTR-TORT	1.367 ± 0.068	1.359 ± 0.062	1.363 ± 0.060	1.386 ± 0.089	0.515	0.562
UWF-Df	1.422 ± 0.097	1.440 ± 0.099	1.454 ± 0.070	1.340 ± 0.082	0.002**	0.006^aa^
UWF-TORT	1.401 ± 0.070	1.401 ± 0.056	1.430 ± 0.079	1.360 ± 0.062	0.026*	0.053

No-DR, no diabetic retinopathy; NPDR, nonproliferative diabetic retinopathy; PDR, proliferative; BH−FDR, Benjamini−Hochberg false discovery rate.

*p<0.05 one-way ANOVA.

**RAW p<0.01 one-way ANOVA.

^aa^ Adjusted *p* < 0.01 after BH−FDR correction.

### Baseline RMPs correlations and clinical changes after RASi treatment

Interestingly, the baseline UWF-vein-TORT was decreased, associated with the decline in eGFR (*r* = 0.449, *p* = 0.019) and the increase in proteinuria assessed by PCR and ACR (*p* = 0.015 and *p* = 0.008). No significant correlations were observed between other RMPs and clinical indicators, as shown in **Table 3**. After 12 weeks of RASi therapy, no significant differences were found in blood pressure, HbA1c, or serum lipid profiles between baseline and post-treatment. PCR decreased significantly (*p* = 0.049) from baseline, without significant changes in other clinical data (**Table 4**).

**Table 3:** Correlation analyses between baseline clinical data and RMPs before renin-angiotensin system inhibitors (RASi) treatment.

Baseline data	SCr, μmol/L		CysC, mg/L		eGFR, mL/min/1.73m^2^			Proteinuria, g/24h			PCR, mg/gCr		ACR, mg/gCr	
	r	*p*	r	*p*	r	*p*	r		*p*	r		*p*	r	*p*
CTR-Artery-*Df*	0.354	0.070	0.085	0.674	-0.046	0.821	-0.093		0.646	-0.147		0.466	-0.199	0.320
CTR-Artery-TORT	0.183	0.361	0.051	0.799	-0.165	0.411	-0.040		0.842	-0.039		0.847	-0.043	0.830
CTR-Vein-*Df*	0.209	0.296	0.229	0.250	-0.078	0.699	0.111		0.580	0.184		0.359	0.174	0.385
CTR-Vein-TORT	0.077	0.704	0.019	0.924	-0.091	0.652	0.076		0.705	-0.010		0.959	0.029	0.887
UWF-Artery-*Df*	0.168	0.402	-0.136	0.497	0.232	0.244	-0.105		0.602	-0.255		0.199	-0.292	0.140
UWF-Artery-TORT	-0.025	0.902	0.174	0.384	0.059	0.769	-0.071		0.723	-0.068		0.737	-0.156	0.436
UWF-Vein-*Df*	0.143	0.476	-0.161	0.423	0.274	0.167	-0.092		0.647	-0.284		0.151	0.306	0.121
UWF-Vein-TORT	0.043	0.832	-0.319	0.105	0.449	0.019^*^	-0.202		0.311	-0.462		0.015^*^	-0.501	0.008^**^
CTR-*Df*	0.294	0.137	0.129	0.521	-0.055	0.784	0.004		0.983	0.047		0.816	0.004	0.986
CTR-TORT	0.153	0.445	0.023	0.910	-0.161	0.423	0.098		0.627	0.113		0.575	0.140	0.485
UWF-*Df*	0.115	0.566	-0.181	0.365	0.300	0.129	-0.104		0.604	-0.244		0.221	-0.269	0.174
UWF-TORT	-0.133	0.509	-0.207	0.300	0.370	0.058	-0.109		0.587	-0.248		0.213	-0.337	0.0865

*p<0.05 Pearson (if normally distributed) or Spearman’s rank (if non-normally distributed) correlation analysis.

**p<0.01 Pearson or Spearman’s rank correlation analysis.

**Table 4:** Changes in clinical data after RASi treatment.

Clinical data	Before RASi treatment	After RASi treatment	Cohen’s d/r	Mean/Median Difference (95% CI)	*P*-value
Systolic Blood Pressure, mmHg	130 ± 7	130 ± 12	0.022	0.26 (-4.34, 4.86)	0.909
Diastolic Blood Pressure, mmHg	81 ± 7	79 ± 9	-0.224	-2.19 (-6.04, 1.67)	0.210
SCr, μmol/L	78.1 ± 17.7	81.2 ± 20.1	0.250	3.14 (-1.83, 8.13)	0.205
CysC, mg/L	1.28 ± 0.33	1.28 ± 0.36	0.006	0.001 (-0.071, 0.073)	0.975
eGFR^a^, mL/min/1.73m^2^	73.7 ± 18.5	73.1 ± 4.11	-0.056	-0.62 (-5.01, 3.77)	0.255
HbA1c, %	7.38 ± 0.74	7.71 ± 1.00	0.392	0.30 (-0.003, 0.61)	0.052
Total Cholesterol, mmol/L	4.72 ± 1.28	4.66 ± 1.48	-0.059	-0.05 (-0.42, 0.31)	0.762
Triglyceride, mmol/L	2.80 ± 2.74	3.11 ± 3.60	0.159	0.31 (-0.46, 1.09)	0.416
PCR, mg/gCr	316(153, 1520)	306(118, 532)	-0.379	-65.00 (-98.00, -11.00)	0.049*
ACR, mg/gCr	235(108, 610)	218(91, 490)	-0.345	-35.00 (-79.00, -5.00)	0.073

*p<0.05 Wilcoxon matched-pairs signed-rank test (non-normally distributed).

### RMPs changes after RASi treatment

**Figure 3:**
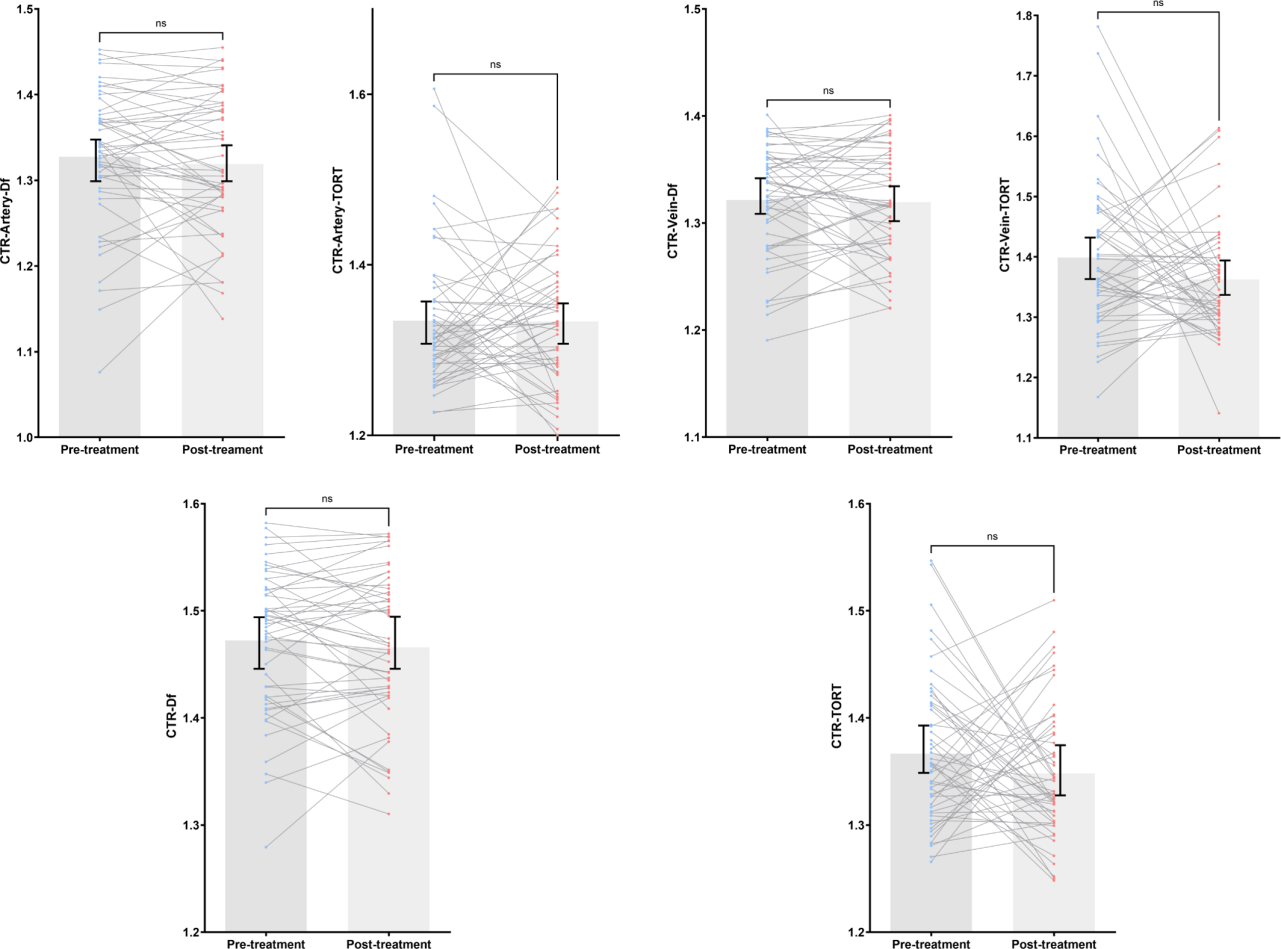
Schematic Diagram of Retinal Microvascular Parameters (RMPs) Changes in the Ultra-Wide-Field (UWF) Images After RASi Administration. Gray bars represented the mean values, and black dotted lines indicated the 95% confidence intervals (95% CI).

**Figure 4:**
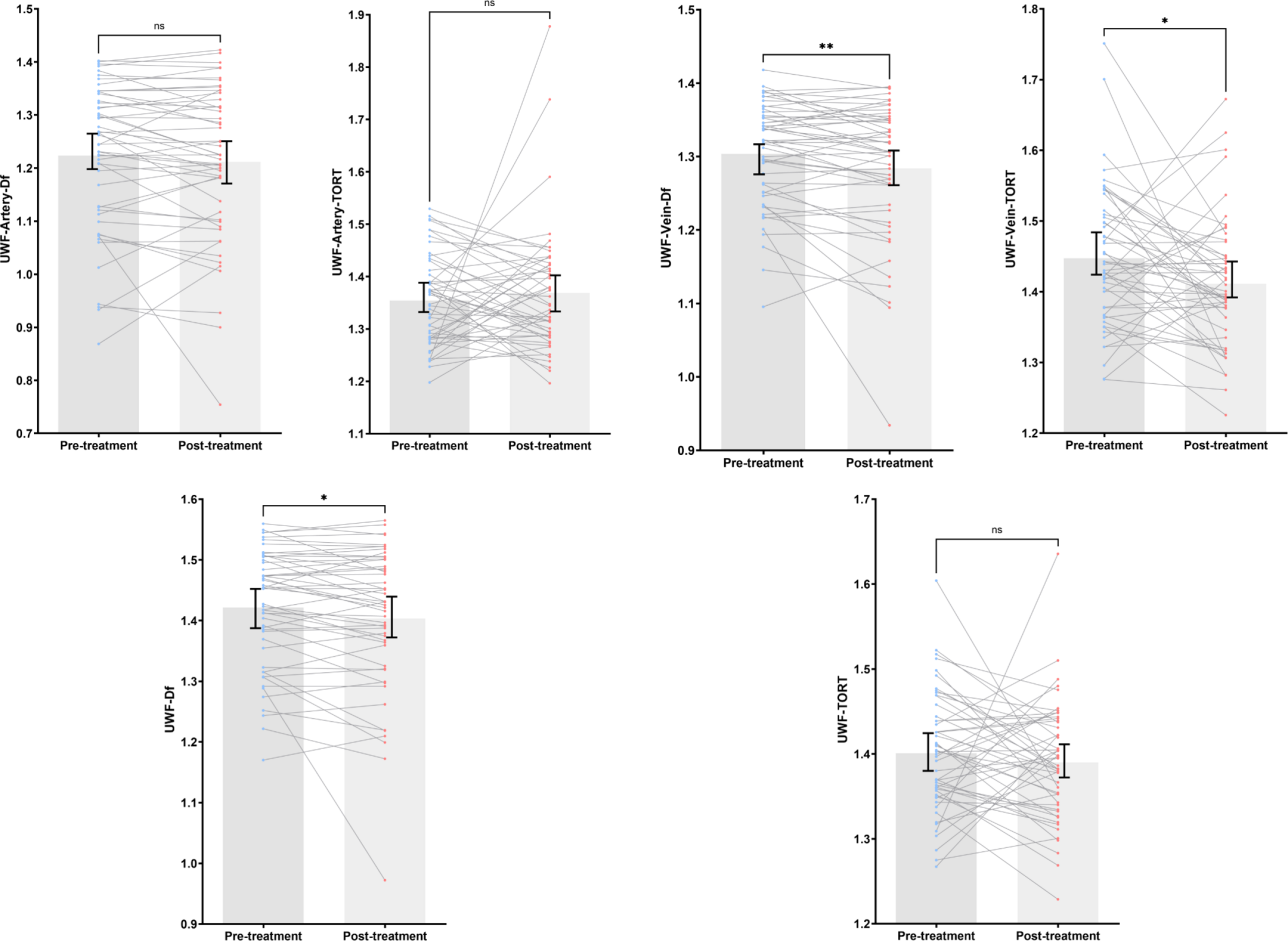
Schematic Diagram of Retinal Microvascular Parameters (RMPs) Changes in the Central Region (CTR) After RASi Administration. Gray bars represented the mean values, and black dotted lines indicated the 95% confidence intervals (95% CI).

Fundus examinations before and after 12 weeks of RASi treatment showed a significant decrease in UWF-vein-D*f* (adjusted *p* = 0.0389) (**Table 5**; **Figure 3**). No significant changes were observed in all the CTR vascular parameters after RASi treatment (**Table 5**; **Figure 4**). In patients with milder fundus lesions of both the No-DR and NPDR groups, UWF fundus images revealed significant decreases in UWF-vein-TORT (adjusted *p* = 0.0496) (**Table 6**).

**Table 5:** Changes in RMPs after RASi treatment. .

Retinal vascular metrics	Before RASi treatment	After RASi treatment	Cohen’s d	Mean Difference (95% CI)	RAW p-value	BH-FDR adjusted p-value
CTR-Artery-*Df*	1.328 ± 0.080	1.319 ± 0.079	-0.141	-0.009 (-0.029, 0.012)	0.4144	0.8288
CTR-Artery-TORT	1.335 ± 0.080	1.334 ± 0.075	-0.008	-0.001 (-0.030, 0.028)	0.9547	0.9547
CTR-Vein-*Df*	1.322 ± 0.050	1.319 ± 0.051	-0.044	-0.002 (-0.017, 0.013)	0.7920	0.7920
CTR-Vein-TORT	1.399 ± 0.122	1.363 ± 0.094	-0.232	-0.036 (-0.078, 0.006)	0.0945	0.1889
UWF-Artery-*Df*	1.223 ± 0.136	1.212 ± 0.144	-0.172	-0.012 (-0.035, 0.011)	0.3106	0.4659
UWF-Artery-TORT	1.354 ± 0.086	1.369 ± 0.121	0.101	0.014 (-0.024, 0.053)	0.4659	0.4659
UWF-Vein-*Df*	1.304 ± 0.071	1.284 ± 0.093	-0.384	-0.020 (-0.036, -0.004)	0.0195*	0.0389^a^
UWF-Vein-TORT	1.447 ± 0.098	1.411 ± 0.091	-0.307	-0.036 (-0.072, 0)	0.0542	0.0542
CTR-*Df*	1.472 ± 0.066	1.466 ± 0.070	-0.136	-0.006 (-0.023, 0.011)	0.4642	0.4642
CTR-TORT	1.367 ± 0.068	1.348 ± 0.061	-0.195	-0.018 (-0.043, 0.006)	0.1493	0.2986
UWF-*Df*	1.422 ± 0.097	1.403 ± 0.118	-0.325	-0.018 (-0.037, 0)	0.0600	0.1200
UWF-TORT	1.401 ± 0.070	1.390 ± 0.069	-0.119	-0.011 (-0.037, 0.015)	0.4183	0.4183

*RAW p<0.05 Linear mixed-effects model with patient ID as a random intercept.

^a^ Adjusted *p* < 0.05 after BH−FDR correction.

**Table 6:** Changes in RMPs of No-DR and NPDR patients (N = 40) after RASi treatment.

Retinal vascular metrics	Before RASi treatment	After RASi treatment	Cohen’s d	Mean Difference (95% CI)	RAW p-value	BH-FDR adjusted p-value
CTR-Artery-*Df*	1.354 ± 0.058	1.336 ± 0.078	-0.298	-0.017 (-0.039, 0.005)	0.1339	0.2679
CTR-Artery-TORT	1.335 ± 0.077	1.342 ± 0.076	0.065	0.007 (-0.026, 0.040)	0.6770	0.6770
CTR-Vein-*Df*	1.327 ± 0.049	1.330 ± 0.048	0.080	0.004 (-0.013, 0.021)	0.6635	0.6635
CTR-Vein-TORT	1.387 ± 0.113	1.376 ± 0.086	-0.077	-0.011 (-0.055, 0.033)	0.6353	0.6635
UWF-Artery- *Df*	1.267 ± 0.109	1.243 ± 0.143	-0.367	-0.024 (-0.050, 0.001)	0.0693	0.1387
UWF-Artery- TORT	1.371 ± 0.079	1.373 ± 0.102	0.012	0.001 (-0.036, 0.039)	0.9399	0.9399
UWF-Vein- *Df*	1.317 ± 0.067	1.299 ± 0.094	-0.317	-0.018 (-0.037, 0.001)	0.0716	0.0716
UWF-Vein-TORT	1.455 ± 0.091	1.411 ± 0.084	-0.391	-0.044 (-0.081, -0.007)	0.0248*	0.0496^a^
CTR-*Df*	1.490 ± 0.057	1.482 ± 0.067	-0.169	-0.008 (-0.027, 0.011)	0.4168	0.8335
CTR-TORT	1.361 ± 0.060	1.359 ± 0.059	-0.021	-0.002 (-0.028, 0.024)	0.8918	0.8918
UWF-*Df*	1.446 ± 0.087	1.426 ± 0.119	-0.334	-0.021 (-0.043, -0.001)	0.0716	0.1285
UWF-TORT	1.413 ± 0.068	1.392 ± 0.059	-0.270	-0.021 (-0.048, 0.006)	0.1285	0.1285

*RAW p<0.05 Linear mixed-effects model with patient ID as a random intercept.

^a^ Adjusted *p* < 0.05 after BH−FDR correction.

### Correlation between changes in RMPs and clinical indicators (ΔKidney) after RASi treatment

After RASi treatment, a significant positive correlation between the change in Cr (ΔCr) and UWF-vein-TORT (ΔTORT) was observed (*r* = 0.413, *p* = 0.032 in **Table 7**). A multivariable linear regression model showed that ΔTORT was not independently associated with ΔCr after adjusting for age, sex, baseline Cr, and baseline UWF-vein-TORT (β=62.466, 95% CI: -11.369 to 136.301, *p*=0.093). The overall model explained only 4.1% of the variance in ΔCr (adjusted R²=0.041, *p*=0.309).

**Table 7:** Correlation analyses between changes in clinical data and alterations in RMPs after RASi treatment.

Baseline data	ΔSCr, μmol/L		ΔCysC, mg/L		ΔeGFR, mL/min/1.73m^2^			ΔPCR, mg/gCr		ΔACR, mg/gCr	
	r	*p*	r	*p*	r		*p*	r	*p*	r	*p*
**Δ**CTR**-**Artery-*Df*	-0.067	0.739	0.316	0.108	0.158	0.433		0.082	0.683	-0.030	0.883
**Δ**CTR-Artery-TORT	-0.048	0.814	-0.148	0.461	0.285	0.149		0.086	0.669	0.064	0.751
**Δ**CTR-Vein-*Df*	-0.120	0.551	-0.041	0.839	0.051	0.799		-0.024	0.904	-0.164	0.413
**Δ**CTR-Vein-TORT	0.164	0.413	0.257	0.196	-0.256	0.198		0.190	0.341	0.172	0.391
**Δ**UWF-Artery-*Df*	0.127	0.527	0.346	0.077	-0.166	0.408		0.068	0.735	-0.031	0.880
**Δ**UWF-Artery-TORT	0.282	0.154	0.271	0.171	-0.158	0.433		0.073	0.716	0.087	0.667
**Δ**UWF-Vein-*Df*	0.242	0.224	0.016	0.936	-0.164	0.413		0.130	0.517	0.045	0.825
**Δ**UWF-Vein-TORT	0.413	0.032^*^	0.219	0.273	-0.363	0.063		-0.173	0.389	-0.197	0.324
**Δ**CTR-*Df*	0.128	0.525	0.137	0.495	-0.055	0.785		-0.072	0.721	-0.197	0.325
**Δ**CTR-TORT	0.243	0.222	0.139	0.489	-0.162	0.419		0.199	0.320	0.172	0.392
**Δ**UWF-*Df*	0.317	0.108	0.081	0.689	-0.210	0.293		0.137	0.496	0.043	0.832
**Δ**UWF-TORT	0.037	0.856	0.109	0.589	-0.059	0.772		0.248	0.211	0.238	0.231

*p<0.05 Spearman’s rank correlation.

## Discussion

In this pilot study, using AI-based analysis of UWF images, we are the first to demonstrate that RASi therapy altered RMPs, particularly within the peripheral venous network, consistent with the decrease in proteinuria. Moreover, the decrease in UWF venous tortuosity was significantly correlated with the reduction in Cr. UWF venous tortuosity was also paralleled by a decline in eGFR (*r* = 0.449, *p* = 0.019) and an increase in proteinuria. It first provides preliminary evidence for the RASi treatment effect in the fundus of DKD patients.

This study is the first to investigate changes in fundus vasculature following RASi treatment in patients with DKD using an AI analysis model. Both DKD and DR are microvascular complications of DM (2), and the fundus lesions were believed to be a window for assessing diabetic kidney involvement (5). The protective effect of RASi on DKD has been demonstrated as a milestone and a fundamental therapy (20). A local RAS also exists in the eye (21), and the protective effect of RASi on DR has been demonstrated in numerous clinical and basic studies (22). The possible mechanisms include attenuating vascular proliferation and permeability, inhibiting oxidative stress, and alleviating pro-inflammatory responses in endothelial cells. However, it still lacks fundus vasculature data from DKD patients following RASi therapy due to technical limitations. Recently, advances in AI have enabled more sophisticated, multidimensional assessment of fundus images (23), allowing the acquisition of additional peripheral vascular information (17, 24). Our AI analysis model, previously established and validated in several studies (17, 24, 25), successfully identified the effects of RASi on the fundus microvasculature in DKD patients.

In this study, short-term RASi treatment resulted in significant decreases in UWF-Vein-D*f* and UWF-Vein-TORT, without any alteration in the CTR, indicating that retinal peripheral vessels are more sensitive to pharmacotherapy. This spatially heterogeneous pattern of treatment response is consistent with the physiology of retinal blood flow and the pathological characteristics of DR (26). Since the peripheral retinal vessels belong to the terminal circulation, they might be more vulnerable to metabolic and hemodynamic abnormalities and often represent the initial site of pathophysiological alterations in DR. Therefore, this region may exhibit an earlier and more pronounced morphological response to therapeutic interventions.

The D*f* and TORT of fundus vessels are core quantitative indicators for describing the geometric morphology of retinal microcirculation, quantifying the complex architecture and trajectory of the fundus vascular network, and enabling objective statistical evaluation of microvascular changes (27). Although several studies have focused on the D*f* and TORT during the progression of DR, the conclusions remain inconsistent due to differences in the types and regions of vessels involved (28–30). In a 10-year prospective follow-up study conducted by Forster (30), the increased retinal venous TORT was independently associated with DR incidence and could enhance the discriminative ability of DR risk prediction models. A retrospective study also confirmed that venous TORT was significantly higher across all DR groups than in the non-DR group, with the greatest elevation observed in the mild NPDR group (28). Consistent with clinical observations, preclinical animal studies demonstrated that early DR exhibited a sequential pathological alteration, in which vascular injury, hypoxia, and vascular endothelial growth factor (VEGF) activation collectively formed a vicious cycle that drove disease progression (31). Additional animal investigations further revealed that oxidative stress served as a key mediator of vascular tortuosity during DR, which could drive vascular tortuosity through activation of the transforming growth factor-β (TGF-β) pathway and remodeling of the vascular elastic lamina (32). We hypothesized that the elevation of venous TORT in our study may be associated with hypoxia, changes in shear stress, vascular remodeling, and endothelial dysfunction mediated by VEGF and inflammatory factors (IL-6, IL-8) during DR progression, which served as an autoregulatory response of blood vessels to increased hydrostatic pressure (33). We found that UWF-vein-TORT decreased after RASi treatment, suggesting that pharmacological intervention reversed the pathological trend of venous TORT elevation. Regarding D*f*, several previous studies indicated that it decreased in DR patients and correlates with lesion severity (28–30). In addition, a study found that the decrease in D*f* of retinal vessels in diabetic patients was significantly associated with pathologically confirmed diabetic nephropathy (34). This is generally interpreted as capillary loss and a reduction in vascular complexity accompanying DR progression. But in a landmark study by Daxer in 1993 (35). D*f* was significantly increased during the early stage of neovascularization, but decreased markedly due to subsequent capillary occlusion with advanced retinal lesions. Our colleagues using an alternative deep learning-based AI model to assess DR also identified a biphasic trend in retinal vascular D*f* (36): significantly elevated in the NPDR group, but decreased in the severe NPDR and PDR groups. These seemingly contradictory findings suggest that, during the progression of DR, changes in fundus vascular D*f* may initially increase, then decrease. In our study, we observed a decrease in UWF-Vein-D*f* after treatment, suggesting a distinct pathophysiological mechanism. This decrease in D*f* may also indicate the reversal of pathological vascular remodeling. Notably, most current studies have used CTR images and failed to distinguish between arterial and venous vessels, highlighting the need for more refined, well-designed clinical studies to further validate these observations.

When analyzing the correlation between changes in RMPs and alterations in clinical data, we found that the decrease in UWF-Vein-TORT was positively correlated with the reduction in Cr. However, in the subsequent multiple linear regression model, the independent association between ΔUWF-Vein-TORT and ΔCr was no longer statistically significant. Notably, the adjusted coefficient of determination of the regression model was low (adjusted R²=0.041), indicating limited explanatory power of the model for outcome variation. Accordingly, these findings should be interpreted cautiously. This result indicates that the synchronous changes during treatment mainly reflect their dependence on the severity of the common baseline disease rather than a direct and independent pathophysiological link. In addition, the relatively limited sample size may have reduced the statistical power to detect small independent effects after adjusting for multiple variables, and further validation with an expanded sample size is required in future studies. These findings suggested that RASi-induced RMPs alterations could potentially serve as a non-invasive surrogate for monitoring intrarenal hemodynamic responses, perhaps helping to guide treatment individualization. Moreover, retinal vascular assessment could be integrated into routine screening for early DKD progression, given its correlation with renal microvascular injury.

Our study has several limitations. First, this study is a pilot study with a small sample size, which not only limits the generalizability of the results, but also reduces the statistical power of multivariate regression analyses. In addition, the absence of a control group limits causal inference. Second, subgroup analyses were constrained by insufficient sample size, which may compromise the reliability of subgroup−specific outcomes. Third, the follow-up duration of this study was relatively short, limiting the evaluation of the association between RMPs and long-term clinical outcomes. Fourth, although the AI model employed in our study has been extensively validated in prior literature, it is not exempt from the inherent biases universal to all machine learning systems. Fifth, potential confounding from concomitant medications existed. To minimize this bias, we enforced a strict medication protocol requiring discontinuation of RASi for at least 4 weeks before enrollment to eliminate their short-term impacts on retinal vascular parameters. Last but not least, the underlying pathophysiological mechanisms remain speculative in the present study; we did not conduct direct mechanistic validation experiments to confirm causal pathways. In the future, large−sample, long−term randomized controlled trials, combined with mechanistic investigations, are required to verify our preliminary correlational findings and clarify potential biological mechanisms.

In conclusion, we first identified significant changes in the retinal peripheral venous network following a short-term RASi treatment using a validated deep learning AI model and provided potential associative clues for novel therapeutic targets and clinical biomarkers in patients with diabetes mellitus.

## Data Availability

The original contributions presented in the study are included in the article/**Supplementary Material**. Further inquiries can be directed to the corresponding authors.

## Supplementary material

Supplementary Table 1 Intergroup comparison of significantly different baseline RMPs across patients with different grades of DR. Mean difference=first group means minus second group means; negative values indicate the first group has a lower mean. ^**^Adjusted *p* < 0.01 in pairwise comparisons with Tukey’s HSD *post hoc* tests for multiple testing.

## References

[1] American Diabetes Association Professional Practice Committee for Diabetes . Chronic kidney disease and risk management: Standards of care in diabetes-2026. Diabetes Care. (2026) 49:S246–60. doi: 10.2337/dc26-s011 41358881 PMC12690176

[2] American Diabetes Association Professional Practice Committee for Diabetes . Retinopathy, neuropathy, and foot care: Standards of care in diabetes-2026. Diabetes Care. (2026) 49:S261–76. doi: 10.2337/9781580408387.ch19 PMC12690177 41358886

[3] Duan J Liu D Zhao Z Liang L Pan S Tian F . Short-term duration of diabetic retinopathy as a predictor for development of diabetic kidney disease. J Transl Int Med. (2023) 11:449–58. doi: 10.2478/jtim-2022-0074 38130638 PMC10732346

[4] Tang S An X Sun W Zhang Y Yang C Kang X . Parallelism and non-parallelism in diabetic nephropathy and diabetic retinopathy. Front Endocrinol (Lausanne). (2024) 15:1336123. doi: 10.3389/fendo.2024.1336123 38419958 PMC10899692

[5] D'Souza YB Short CD . The eye--a window on the kidney. Nephrol Dial Transplant. (2009) 24:3582–4. doi: 10.1007/978-3-030-22696-1_8 19679560

[6] Lewis EJ Hunsicker LG Bain RP Rohde RD . The effect of angiotensin-converting-enzyme inhibition on diabetic nephropathy. The Collaborative Study Group. N Engl J Med. (1993) 329:1456–62. doi: 10.1056/nejm199311113292004 8413456

[7] Brenner BM Cooper ME de Zeeuw D Keane WF Mitch WE Parving HH . Effects of losartan on renal and cardiovascular outcomes in patients with type 2 diabetes and nephropathy. N Engl J Med. (2001) 345:861–9. doi: 10.1016/s1062-1458(01)00549-9 11565518

[8] Lewis EJ Hunsicker LG Clarke WR Berl T Pohl MA Lewis JB . Renoprotective effect of the angiotensin-receptor antagonist irbesartan in patients with nephropathy due to type 2 diabetes. N Engl J Med. (2001) 345:851–60. doi: 10.1056/nejmoa011303 11565517

[9] Davis KN Hines AE Schaefer MC Naseman KW . Protecting the kidneys: Update on therapies to treat diabetic nephropathy. Clin Diabetes. (2022) 40:305–11. doi: 10.2337/cd21-0090 35983418 PMC9331620

[10] Group UPDSU . Intensive blood-glucose control with sulphonylureas or insulin compared with conventional treatment and risk of complications in patients with type 2 diabetes (UKPDS 33). UK Prospective Diabetes Study (UKPDS) Group. Lancet. (1998) 352:837–53. doi: 10.1007/bf00400359 9742976

[11] Chaturvedi N Sjolie AK Stephenson JM Abrahamian H Keipes M Castellarin A . Effect of lisinopril on progression of retinopathy in normotensive people with type 1 diabetes. The EUCLID Study Group. EURODIAB Controlled Trial of Lisinopril in Insulin-Dependent Diabetes Mellitus. Lancet. (1998) 351:28–31. doi: 10.1016/s0140-6736(97)06209-0 9433426

[12] Wang T Li H Wang C Li X Deng A Jiao X . Diabetic retinopathy as a sentinel of systemic vascular dysfunction: Shared molecular mechanisms with cardiovascular disease. Exp Eye Res. (2025) 261:110644. doi: 10.1016/j.exer.2025.110644 40972855

[13] Ying X Freedland KE Powell LH Stuart EA Ehrhardt S Mayo-Wilson E . Determining sample size for pilot trials: a tutorial. Bmj. (2025) 390:e083405. doi: 10.1136/bmj-2024-083405 40780848

[14] Totton N Lin J Julious S Chowdhury M Brand A . A review of sample sizes for UK pilot and feasibility studies on the ISRCTN registry from 2013 to 2020. Pilot Feasibility Stud. (2023) 9(1):188. doi: 10.1186/s40814-023-01416-w 37990337 PMC10662929

[15] Das T Padakandla SR Shivaji S Jayasudha R Takkar B . Intraocular microbiome in diabetes and diabetic retinopathy: A pilot study. Ophthalmol Ther. (2023) 12:1109–26. doi: 10.1007/s40123-023-00660-w 36719607 PMC10011241

[16] Rovner BW Casten RJ . Trust and glycemic control in black patients with diabetic retinopathy: A pilot study. Diabetes Spectr. (2019) 32:152–5. doi: 10.2337/ds18-0037 31168287 PMC6528393

[17] Zhao X Gu X Meng L Chen Y Zhao Q Cheng S . Screening chronic kidney disease through deep learning utilizing ultra-wide-field fundus images. NPJ Digital Med. (2024) 7(1):275. doi: 10.1038/s41746-024-01271-w 39375513 PMC11458603

[18] Inker LA Eneanya ND Coresh J Tighiouart H Wang D Sang Y . New creatinine- and cystatin C-based equations to estimate GFR without race. N Engl J Med. (2021) 385:1737–49. doi: 10.1056/nejmoa2102953 34554658 PMC8822996

[19] Zhou Z Siddiquee MMR Tajbakhsh N Liang J . UNet++: Redesigning skip connections to exploit multiscale features in image segmentation. IEEE Trans Med Imaging. (2020) 39:1856–67. doi: 10.1109/tmi.2019.2959609 31841402 PMC7357299

[20] Navaneethan SD Zoungas S Caramori ML Chan JCN Heerspink HJL Hurst C . Diabetes management in chronic kidney disease: Synopsis of the KDIGO 2022 clinical practice guideline update. Ann Intern Med. (2023) 176:381–7. doi: 10.7326/m22-2904 36623286

[21] Ola MS Alhomida AS Ferrario CM Ahmad S . Role of tissue renin-angiotensin system and the chymase/angiotensin-(1-12) axis in the pathogenesis of diabetic retinopathy. Curr Med Chem. (2017) 24:3104–14. doi: 10.2174/0929867324666170407141955 28403787 PMC5815313

[22] Li X Fu Y-H Tong X-W Zhang Y-T Shan Y-Y Xu Y-X . RAAS in diabetic retinopathy: Mechanisms and therapies. Arch Endocrinol Metab. (2024) 68:e230292. doi: 10.20945/2359-4292-2023-0292 38652701 PMC11081058

[23] Yang Q Bee YM Lim CC Sabanayagam C Yim-Lui Cheung C Wong TY . Use of artificial intelligence with retinal imaging in screening for diabetes-associated complications: Systematic review. EClinicalMedicine. (2025) 81:103089. doi: 10.1016/j.eclinm.2025.103089 40052065 PMC11883405

[24] Zhao Q Wang C Meng L Cheng S Gu X Chen Y . Central and peripheral changes in the retina and choroid in patients with diabetes mellitus without clinical diabetic retinopathy assessed by ultra-wide-field optical coherence tomography angiography. Front Public Health. (2023) 11. doi: 10.3389/fpubh.2023.1194320 37383256 PMC10293646

[25] Zhao X Liu Y Zhang W Meng L Lv B Lv C . Relationships between retinal vascular characteristics and renal function in patients with type 2 diabetes mellitus. Trans Vision Sci Technol. (2021) 10(2):20. doi: 10.1167/tvst.10.2.20 34003905 PMC7884293

[26] Kusuhara S Fukushima Y Ogura S Inoue N Uemura A . Pathophysiology of diabetic retinopathy: The old and the new. Diabetes Metab J. (2018) 42:364–76. doi: 10.4093/dmj.2018.0182 30362302 PMC6202564

[27] Gao Y Xu L He N Ding Y Zhao W Meng T . A narrative review of retinal vascular parameters and the applications (Part I): Measuring methods. Brain Circ. (2023) 9:121–8. doi: 10.4103/bc.bc_8_23 38020955 PMC10679626

[28] Fathimah FSN Ari Widjaja S Sasono W Yustiarini I Firmansjah M Prakosa AD . Retinal vessel tortuosity and fractal dimension in diabetic retinopathy. Int J Retina Vitreous. (2025) 11(1):64. doi: 10.1186/s40942-025-00688-z 40506774 PMC12164056

[29] Zhang J Ma K Luo Z Wang G Feng Z Huang Y . Combining functional and morphological retinal vascular characteristics achieves high-precision diagnosis of mild non-proliferative diabetic retinopathy. J Transl Med. (2024) 22:798. doi: 10.1186/s12967-024-05597-7 39198867 PMC11360493

[30] Forster RB Garcia ES Sluiman AJ Grecian SM McLachlan S MacGillivray TJ . Retinal venular tortuosity and fractal dimension predict incident retinopathy in adults with type 2 diabetes: The Edinburgh Type 2 Diabetes Study. Diabetologia. (2021) 64:1103–12. doi: 10.1007/s00125-021-05388-5 33515071 PMC8012328

[31] Yamato M Kato N Yamada KI Inoguchi T . The early pathogenesis of diabetic retinopathy and its attenuation by sodium-glucose transporter 2 inhibitors. Diabetes. (2024) 73:1153–66. doi: 10.2337/figshare.25577400.v1 PMC11208076 38608284

[32] Fumoto T Kinoshita S Sasaki T Shimamura N Ohkuma H . Oxidative stress mediates vascular tortuosity. Antioxidants (Basel). (2021) 10(6):926. doi: 10.3390/antiox10060926 34200411 PMC8228074

[33] Vilela MA Amaral CE Ferreira MAT . Retinal vascular tortuosity: Mechanisms and measurements. Eur J Ophthalmol. (2021) 31:1497–506. doi: 10.1177/1120672120979907 33307777

[34] Liu F Chen X Wang Q Lin W Li Y Zhang R . Correlation between retinal vascular geometric parameters and pathologically diagnosed type 2 diabetic nephropathy. Clin Kidney J. (2024) 17:sfae204. doi: 10.1093/ckj/sfae204 39099565 PMC11292218

[35] Daxer A . Characterisation of the neovascularisation process in diabetic retinopathy by means of fractal geometry: Diagnostic implications. Graefes Arch Clin Exp Ophthalmol. (1993) 231:681–6. doi: 10.1007/bf00919281 8299974

[36] Xiao X Zhao J Lin S Yang Y Li W Zhou Y . Relationships between retinal vascular characteristics and systemic indicators in patients with diabetes mellitus. Invest Ophthalmol Visual Sci. (2025) 66(4):72. doi: 10.1167/iovs.66.4.72 40272369 PMC12032846

