# Supplementary material for: Effect of RAS inhibitors on retinal microvasculature in DKD using AI-based ultra-wide-field images
Source: Front Endocrinol (Lausanne). 2026 Jun 26;17:1840180. doi: 10.3389/fendo.2026.1840180 (PMC13349828; doi:10.3389/fendo.2026.1840180)
Supplement: Supplementary Table 1 — Intergroup comparison of significantly different baseline RMPs across patients with different grades of DR. Mean difference=first group means minus second group means; negative values indicate the first group has a lower mean. **Adjusted p < 0.01 in pairwise comparisons with Tukey’s HSD post hoc tests for multiple testing. [file Table1.pdf]

Supplemental Table 1. Intergroup comparison of significantly different baseline RMPs across patients with different grades of DR.

**Retinal vascular metrics**	**Comparison**	**Mean difference (95%CI)**	***p*-value**
**CTR-Artery-*Df***	No-DR vs. NPDR	0.006 (-0.043, 0.056)	0.947
	No-DR vs. PDR	0.116 (0.060, 0.172)	$< 0.001^{**}$
	NPDR vs. PDR	0.109 (0.051, 0.168)	$< 0.001^{**}$
**UWF-Artery-*Df***	No-DR vs. NPDR	-0.022 (-0.109, 0.065)	0.816
	No-DR vs. PDR	0.178 (0.082, 0.275)	$< 0.001^{**}$
	NPDR vs. PDR	0.200 (0.098, 0.302)	$< 0.001^{**}$
**CTR-*Df***	No-DR vs. NPDR	0.007 (-0.038, 0.052)	0.924
	No-DR vs. PDR	0.079 (0.028, 0.129)	0.001^{**}
	NPDR vs. PDR	0.072 (0.018, 0.125)	0.006^{**}
**UWF-*Df***	No-DR vs. NPDR	-0.015 (-0.082, 0.052)	0.856
	No-DR vs. PDR	0.100 (0.025, 0.174)	0.006^{**}
	NPDR vs. PDR	0.115 (0.035, 0.194)	0.003^{**}

Mean difference=first group means minus second group means; negative values indicate the first group has a lower mean.

**Adjusted $p<0.01$ in pairwise comparisons with Tukey's HSD post hoc tests for multiple testing.
